# Potential role of endoplasmic reticulum stress in doxorubicin-induced cardiotoxicity-an update

**DOI:** 10.3389/fphar.2024.1415108

**Published:** 2024-08-12

**Authors:** Mingli Sun, Xin Zhang, Boxuan Tan, Qingya Zhang, Xiaopeng Zhao, Dan Dong

**Affiliations:** ^1^ College of Exercise and Health, Shenyang Sport University, Shenyang, Liaoning, China; ^2^ College of Basic Medical Science, China Medical University, Shenyang, Liaoning, China; ^3^ Innovation Institute, China Medical University, Shenyang, Liaoning, China

**Keywords:** endoplasmic reticulum stress, doxorubicin-induced cardiotoxicity, apoptosis, autophagy, targeted therapy

## Abstract

As a chemotherapy agent, doxorubicin is used to combat cancer. However, cardiotoxicity has limited its use. The existing strategies fail to eliminate doxorubicin-induced cardiotoxicity, and an in-depth exploration of its pathogenesis is in urgent need to address the issue. Endoplasmic reticulum stress (ERS) occurs when Endoplasmic Reticulum (ER) dysfunction results in the accumulation of unfolded or misfolded proteins. Adaptive ERS helps regulate protein synthesis to maintain cellular homeostasis, while prolonged ERS stimulation may induce cell apoptosis, leading to dysfunction and damage to tissue and organs. Numerous studies on doxorubicin-induced cardiotoxicity strongly link excessive activation of the ERS to mechanisms including oxidative stress, calcium imbalance, autophagy, ubiquitination, and apoptosis. The researchers also found several clinical drugs, chemical compounds, phytochemicals, and miRNAs inhibited doxorubicin-induced cardiotoxicity by targeting ERS. The present review aims to outline the interactions between ERS and other mechanisms in doxorubicin-induced cardiotoxicity and summarize ERS’s role in this type of cardiotoxicity. Additionally, the review enumerates several clinical drugs, phytochemicals, chemical compounds, and miRNAs targeting ERS for considering therapeutic regimens that address doxorubicin-induced cardiotoxicity.

## 1 Introduction

**FIGURE 1:**
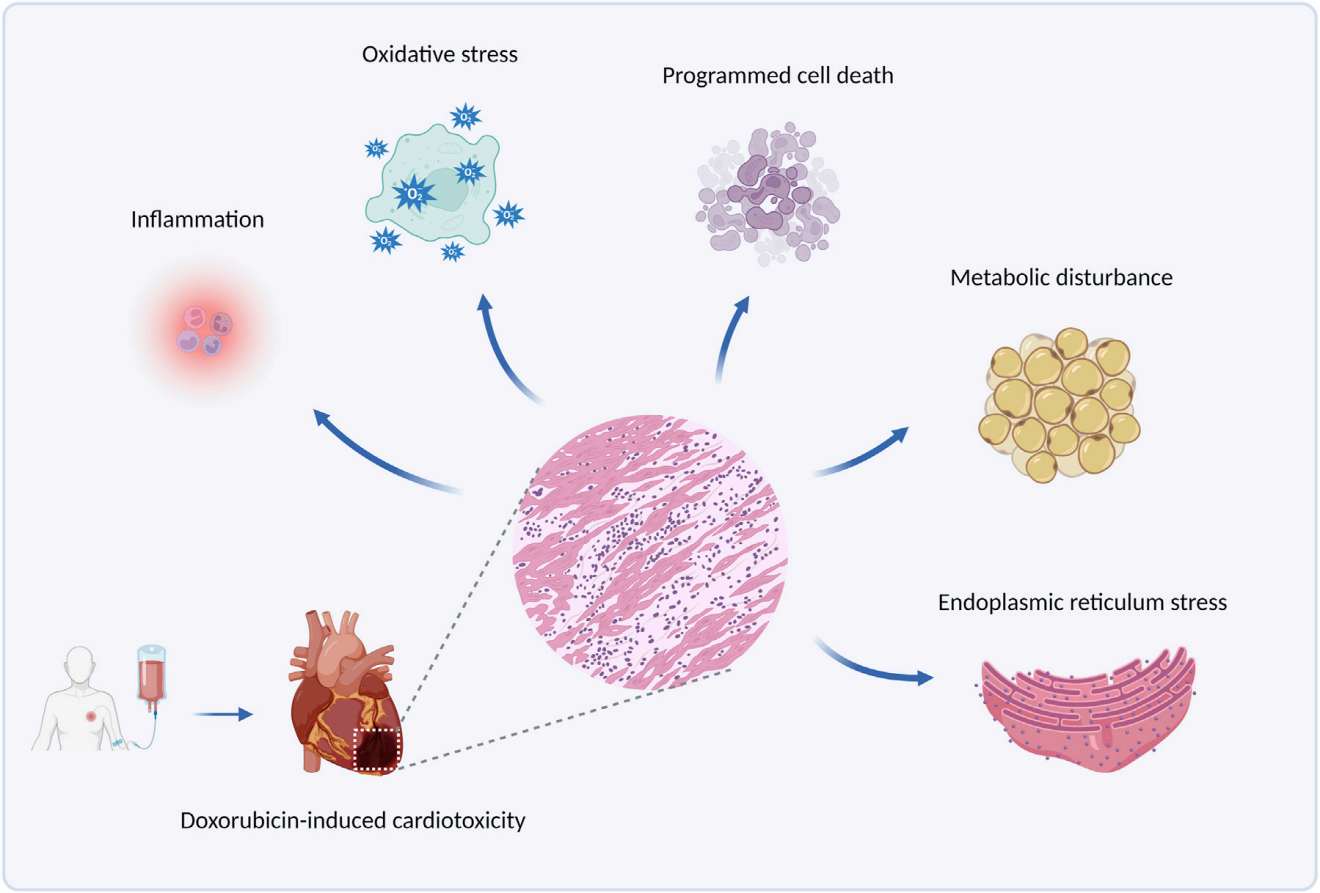
The principal pathological mechanisms underlying doxorubicin-induced cardiotoxicity. Produced using BioRender.com (www.biorender.com). Pathological mechanisms underlying doxorubicin-induced cardiotoxicity involve inflammation, oxidative stress, programmed cell death (such as apoptosis, autophagy, and ferroptosis), metabolic disturbance, endoplasmic reticulum stress, etc.

Doxorubicin (DOX), an anthracycline anticancer drug obtained from the *Streptomyces* Peutius (Damiani et al., 2016), has been widely applied to the treatment of various types of cancer, such as breast cancer, ovarian cancer, and multiple myeloma (Duggan and Keating, 2011). However, cumulative doxorubicin dose exceeding 400–700 mg/m^2^ in adults and 300 mg/m^2^ in children may lead to cardiac damage (Li and Hill, 2014), manifested mainly by arrhythmia, cardiomyopathy, left ventricular dysfunction, and even congestive heart failure, by which its use has been limited. Clinically, echocardiogram, cardiac troponin T, and other cardiac injury-related indicators are detected to diagnose doxorubicin-induced cardiotoxicity (Mitry and Edwards, 2016). Currently, combination therapy of doxorubicin with other anticancer drugs is put into use to alleviate this side effect, and another antioxidant drug, Dexrazoxane, is also beneficial for its prevention (Ludke et al., 2009). Nevertheless, such strategies fail to eliminate doxorubicin-induced cardiotoxicity, and an in-depth exploration of its pathogenesis is in urgent need to address the issue. Some molecular pathological mechanisms, such as inflammation, oxidative stress, programmed cell death, metabolic disturbance, and endoplasmic reticulum stress, etc., are involved in doxorubicin-induced cardiotoxicity (Renu et al., 2018), among which ERS is gradually brought to the forefront. The principal pathological mechanism of doxorubicin-induced cardiotoxicity is illustrated in Figure 1.

Endoplasmic reticulum stress (ERS) is a state of cells under various stimulations that cause unfolded or misfolded proteins to accumulate in the Endoplasmic Reticulum (ER). In the short term, adaptive ERS helps regulate protein synthesis to maintain cellular homeostasis (Yoshida, 2007), while prolonged ERS stimulation may induce cell apoptosis, leading to dysfunction and damage to tissue and organs (Ayaub et al., 2016). ERS is associated with the onset and progression of multiple diseases, including diabetes (Oakes and Papa, 2015), bone disease (Wen et al., 2023), intervertebral disc degeneration (Huang et al., 2022b), and cardiovascular disease (Zhu and Zhou, 2021a). Recent studies have also identified ERS as one of the contributors to doxorubicin-induced cardiotoxicity (Renu et al., 2018; Yarmohammadi et al., 2024), and a review in 2021 summarized the research progress on the role of ERS in doxorubicin-induced cardiotoxicity, providing us with a promising target for its treatment (Yarmohammadi et al., 2021). Subsequently, a growing number of studies investigated the potential role of ERS in doxorubicin-induced cardiotoxicity, so it is necessary for an updated summary of this topic.

In this review, the activation of ERS and its participation in doxorubicin-induced cardiotoxicity via various mechanisms including autophagy, ubiquitination, oxidative stress, calcium homeostasis disorder, and apoptosis will be elaborated in sequence. Moreover, potential drugs, compounds, and miRNAs targeting ERS to address doxorubicin-induced cardiotoxicity will be enumerated, to provide direct evidence for its feasibility.

## 2 ERS and the unfolded protein responses

ER is an essential intracellular protein-processing organelle, whose homeostasis is crucial for normal cellular function (Ellgaard and Helenius, 2003). Under certain conditions, unfolded or misfolded proteins accumulate in ER and induce ERS (Yap et al., 2021). Once ERS happens, the cell activates a process called unfolded protein responses (UPR) to relieve ERS and assist ER back to the state of homeostasis (Malhotra and Kaufman, 2007). UPR consists of three critical ERS receptors: PKR-like ER kinase (PERK), activating transcription factor 6 (ATF6), and inositol-requiring enzyme 1 (IRE1) (Meyerovich et al., 2016). In mammalian cells, ERS activation is regulated by 78-kDa glucose-regulated protein (GRP78), a molecular chaperone in ER (Kimata and Kohno, 2011). Under physiological state, the aforementioned three ERS sensors bind to GRP78 and lose their functions temporarily (Ren et al., 2021). However, the accumulation of unfolded or misfolded proteins promotes the dissociation of GRP78 from ERS sensors and its interaction with unfolded proteins, resulting in the occurrence of UPR (Yap et al., 2021). It has also been suggested that unfolded or misfolded proteins can directly activate IRE1 (Gardner et al., 2013), whereas the activation of ATF6 requires the protein disulfide bond isomerase A5 (PDIA5) (Higa et al., 2014).

PERK is a type I transmembrane protein on ER with a protein kinase structural domain, which is structurally similar to eukaryotic translation-initiation factor 2α (elF2α) kinase (Harding et al., 1999). When dissociation happens between GRP78 and PERK, PERK undergoes homodimerization and trans-phosphorylation, which in turn causes elF2α phosphorylation and inactivation. Inactivation of elF2α inhibits most mRNA translations and protein productions, in which way ERS could be mitigated (Bertolotti et al., 2000; Raven et al., 2008). However, phosphorylated elF2α also upregulates the translation of activating transcription factor 4 (ATF4) (Harding et al., 2003), which turns out to induce apoptosis by increasing the expression of C/EBP homologous protein (CHOP) (Harding et al., 2003; Hu et al., 2018). More than that, ATF4 also enhances elF2α dephosphorylation by increasing GADD34 expression, thereby restoring the impaired translation process (Hu et al., 2018). ATF6 is another transcriptional factor (Glembotski, 2014) with a distinct activation pattern from PERK and IRE1. After the dissociation of ATF6 and GRP78, Golgi localization signalling is activated, allowing ATF6 to enter and obtain its active fragment through the degradation by resident proteases s1 and s2 (Shen et al., 2002). The fragment, ATF6 (p50), enters the nucleus and regulates ERS-related gene transcription (Endres and Reinhardt, 2013). IRE1, another transmembrane protein on ER with the protein kinase and RNase potential (Lee et al., 2008), activates after dissociation with GRP78 and cleaves the mRNA of X-box binding protein 1 (XBP1), resulting in the formation of sheared XBP1 (XBP1s) (Yoshida et al., 2001). Meanwhile, the RNA endonuclease structural domain of IRE1 tears up other mRNAs to destroy them, thus mitigating ERS, a process we call regulated IRE1-dependent decay (RIDD) (Almanza et al., 2022). In addition, IRE1 is capable of regulating cell apoptosis, by not only the activation of CHOPs through the IRE1-XBP1 pathway (Peña and Harris, 2011) but also the awakening of JUK and caspase-12 (Yoneda et al., 2001; Chakrabarti et al., 2011).

**FIGURE 2:**
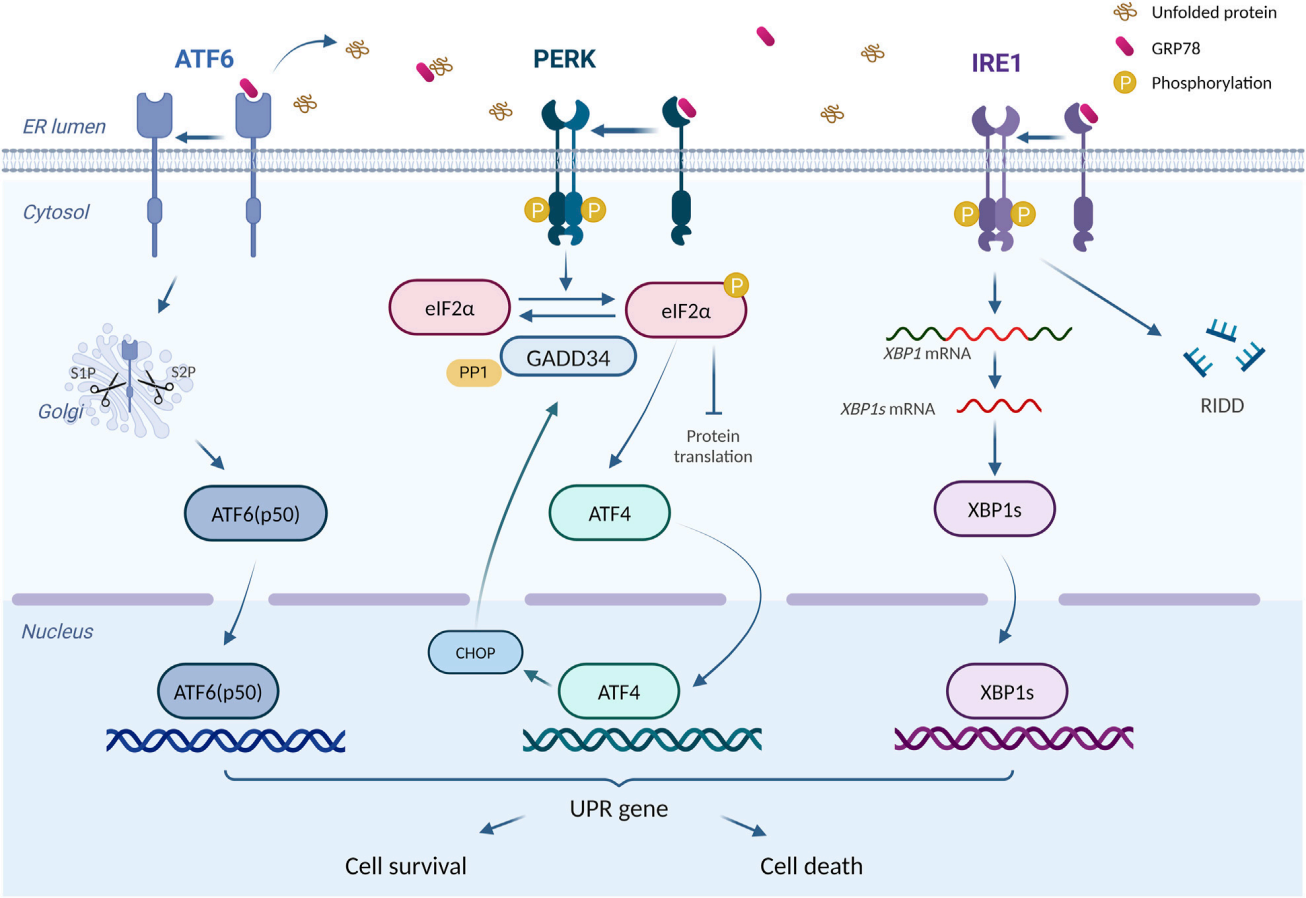
The principal process of UPR response. “UPR Signalling (ATF6, PERK, IRE1)” is produced using BioRender.com (www.biorender.com). Retrieved from https://app.biorender.com/biorender-templates. Dissociates from GRP78, ATF6, IRE1, and PERK trigger the UPR response. Adaptive UPR response restores ER function and cellular homeostasis, whereas excessive ERS leads to cell death.

An adaptive UPR positively works in maintaining normal cellular function (Hetz, 2012). However, prolonged or excessive ERS turns adaptive UPR into a “maladaptive UPR” that drives apoptosis (Hetz et al., 2015). Excessive UPR correlates with various diseases, including diabetes, metabolic disorders, and inflammation (Wang and Kaufman, 2012a). In doxorubicin-induced cardiotoxicity, UPR acts as a double-edged sword. On one hand, maladaptive UPR participates in various pathological processes such as apoptosis, and aggravating doxorubicin-induced cardiac injury (Yarmohammadi et al., 2021). On the other hand, activating adaptive UPR plays a protective role (Maiuolo et al., 2021; Fa et al., 2022). Figure 2 summarizes the principal process of UPR response.

## 3 The role of ERS in doxorubicin-induced cardiotoxicity

**FIGURE 3:**
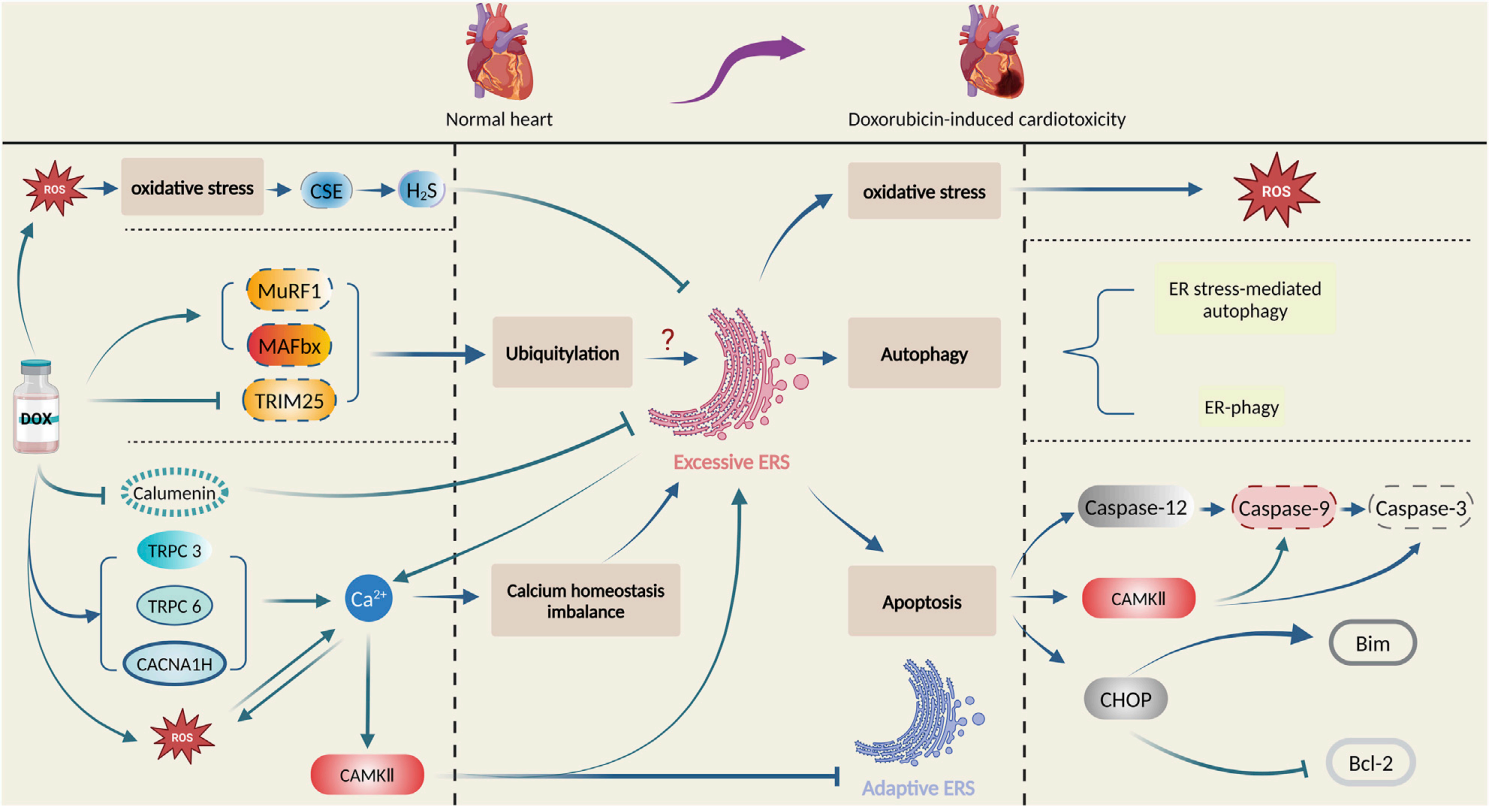
Principal role of ERS in doxorubicin-induced cardiotoxicity. Produced using BioRender.com (www.biorender.com). Doxorubicin promotes oxidative stress, ubiquitination, and calcium homeostasis imbalance, and then activates ERS, which leads to cardiac damage and pathological changes by multiple mechanisms such as oxidative stress, autophagy, and apoptosis.

Numerous factors and intricate mechanisms are involved in the pathogenesis of doxorubicin-induced cardiotoxicity, among which ERS plays a vital role. In this section, the intricate mechanisms of ERS involved in doxorubicin-induced cardiotoxicity will be illustrated. Figure 3 summarizes the principal role of ERS in doxorubicin-induced cardiotoxicity.

### 3.1 ERS and oxidative stress

Increased levels of oxidants in cells may disturb the balance of oxidants and antioxidants, causing an interruption in redox signalling conduction and control as well as molecular damage (Sies, 2015). Oxidative stress is associated with various diseases, including atherosclerosis, chronic obstructive pulmonary disease (COPD), Alzheimer’s disease, and cancer (Forman and Zhang, 2021), also found to be essential in myocardial damage caused by doxorubicin administration (Rawat et al., 2021). Oxidative stress induces ERS (Cubillos-Ruiz et al., 2017), while ERS also promotes reactive oxygen species (ROS) generation, leading to oxidative stress (Zeeshan et al., 2016), indicating a mutual promotion of the two processes. Moreover, they are also inextricably linked to doxorubicin-induced cardiotoxicity. Recently, scientists discovered that doxorubicin-treated cardiomyocytes experienced damage and elevated the quantities of both ROS and ERS-associated proteins, indicating that both oxidative stress and ERS are implicated in doxorubicin-induced cardiotoxicity (Wang et al., 2018e; Kim et al., 2022b). They also discovered that activation of protein arginine methyltransferase 1 (PRMT1) or inhibition of the transient receptor potential ankyrin 1 (TRPA1) channel reduced ROS production and ERS and decreased their harmful effects on doxorubicin-treated cardiomyocytes (Wang et al., 2018e; Kim et al., 2022b). These findings suggest that activating PRMT1 or inhibiting TRPA1 could be potential treatments for doxorubicin-induced cardiotoxicity by targeting oxidative stress and ERS.

Cystathionine-c-lyase (CSE) is one of the key enzymes for H2S synthesis (Filipovic, 2015). Wang et al. (2012b) revealed that doxorubicin treatment led to impaired cardiac function and diminished CSE, increased ROS accumulation, and expression of ERS-associated proteins. However, administration of ROS scavenger attenuated intracellular ROS accumulation, and cardiotoxicity and corrected the activity of CSE. Moreover, H2S markedly suppressed doxorubicin-induced ERS and cardiotoxicity. This suggests that oxidative stress may lead to the inhibition of the CSE-H2S pathway, and induce ERS in doxorubicin-induced cardiotoxicity (Wang et al., 2012b).

Serum and glucocorticoid-regulated protein kinase 1 (SGK1) is a serine-threonine protein kinase from the AGC protein kinase subfamily (Sang et al., 2020). Scientists found increased SGK1 expression attenuated doxorubicin-induced cardiotoxicity in rats and simultaneously inhibited the production of ROS and ERS-associated proteins such as GRP78, while overexpressed GRP78 reverses this inhibitive effect on ROS and cardiotoxicity, suggesting that ERS exacerbates the effects of doxorubicin on cardiomyocyte through enhanced oxidative stress (Wang and Han, 2022b).

In summary, the interaction between oxidative stress and ERS amplified the cardiac injury effect caused by doxorubicin, implying a new treatment for doxorubicin-induced cardiotoxicity through an intervention on oxidative stress and ERS.

### 3.2 ERS and calcium homeostasis imbalance

Calcium ions, key molecules in regulating cardiac electrical and mechanical activities, may sometimes develop homeostasis imbalance in cardiomyocytes. Continuous imbalance in intracellular calcium entry and removal leads to cellular deterioration, resulting in calcium homeostasis imbalance and cell death (Berridge et al., 2003; Vassalle and Lin, 2004). Some calcium-dependent signalling pathways, such as calmodulin kinase, are crucial in the regulation of calcium homeostasis imbalance (Takemoto-Kimura et al., 2017). Imbalances in calcium homeostasis contribute to diverse pathological conditions, including ischemia-reperfusion injury, neurological disorders, pancreatitis, and doxorubicin-induced cardiotoxicity (Calvo-Rodriguez et al., 2020; Li et al., 2021; Petersen et al., 2021; Rawat et al., 2021). Excessive calcium efflux from ER leads to high cytoplasmic calcium ion levels and low levels in ER, followed by ERS induction (Ali and Petrovsky, 2019). Following doxorubicin exposure, intracellular Ca^2+^ concentration of cardiomyocytes is increased, while pretreatment with sarcoplasmic reticulum (SR) Ca^2+^ channel blockers, the Ca^2+^ increase is inhibited, meanwhile, doxorubicin-mediated activation of caspase-3 is significantly inhibited (Kim et al., 2006). In addition, doxorubicin exposure induces calcium-calmodulin (CaM)-dependent protein kinase II (CaMKII) -dependent SR Ca leakage, which partially contributes to cellular calcium homeostasis imbalance (Sag et al., 2011). This indicates that ERS and calcium homeostatic imbalance may be involved in doxorubicin-induced cardiotoxicity.

Altered calcium ion concentration induced by doxorubicin has a complex effect on the role of ERS in cardiomyocytes. Studies have shown that calcium channel proteins may affect ERS and doxorubicin-induced cardiotoxicity via calcium ions. Hu et al. (2019) discovered a significant increase in the expression of Cav3.2(CACNA1H) T-type calcium channel in doxorubicin-induced model rats, along with increased ERS proteins, calcium ion concentration, and developed cardiac dysfunction, while inhibition of CACNA1H channel by ABT-639 resulted in reduced ERS protein level and calcium ion concentration, and recovery of cardiac function (Hu et al., 2019). Transient Receptor Potential (TRP) protein is capable of assembling its subunits C1, C3, C5, and C6 into a calcium channel that regulates intracellular calcium ion concentration (Ohba et al., 2007). In another study, the data show that doxorubicin can disrupt calcium homeostasis and increase ERS levels in cardiomyocytes. Moreover, Salvianolic acid B is responsible for alleviating calcium overload, ERS, and doxorubicin-induced cardiotoxicity by inhibiting TRPC3 and TRPC6 (Chen et al., 2017). These suggest that calcium channel proteins CACNA1H, TRPC3, and TRPC6 may affect ERS via calcium ions in cardiomyocytes following doxorubicin exposure and are expected to become a potential target for doxorubicin-induced cardiotoxicity.

Upon doxorubicin stimulation, calcium-binding protein calumenin is also involved in regulating ERS of cardiomyocytes. Recent research has indicated that there is decreased calumenin level in cardiomyocytes following doxorubicin exposure, and several interventions such as miR378*, astragalus injection, and creatine phosphate disodium salt could inhibit doxorubicin-induced ERS and cardiotoxicity by targeting calumenin (Ma et al., 2016; Wang et al., 2018a; Wang et al., 2018c). These studies indicate that calumenin-regulated calcium homeostasis could alleviate doxorubicin-induced ERS and cardiotoxicity.

CaMKII also regulates ERS in doxorubicin-induced cardiotoxicity. Researchers found Ca^2+^ dependent CaMKII activation is involved in doxorubicin-induced cardiotoxicity by promoting apoptosis, while ERS sensor GRP78 overexpression partly protects cardiomyocytes from doxorubicin-induced cell death by regulating Ca^2+^ dependent pathways (Tscheschner et al., 2019). Scientists also found that CaMKII activation is involved in doxorubicin-induced cardiotoxicity by triggering endoplasmic reticulum stress and apoptosis by regulating the IRE1α/XBP1s pathway (Kong et al., 2022). These have provided evidence that CaMKII might hinder defensive ERS in doxorubicin-induced cardiotoxicity.

There is the ER-mitochondria interconnection in cardiomyocytes following doxorubicin exposure, mediating the interaction of ROS and calcium homeostasis disturbance upon ERS. ERS can be triggered by the dysfunction of mitochondria-associated membranes (MAMs), where Ca^2+^ transduction between the ER and mitochondria takes place (Kim and Choi, 2021). Mitochondria is an important source of ROS during ERS (Cao and Kaufman, 2014). During ERS, there is a vicious cycle between Ca^2+^ release from the ER and mitochondrial ROS production that disrupts cellular homeostasis and induces apoptosis (Csordás and Hajnóczky, 2009). Upon the stimulation by doxorubicin, the intracellular Ca^2+^ concentration of cardiomyocytes is increased, while the Ca^2+^ increase is inhibited by pretreatment with sarcoplasmic reticulum (SR) Ca^2+^ channel blockers and antioxidants. In addition, doxorubicin also induces ROS generation of cardiomyocytes in a time-dependent manner, while ROS production is blocked by the pretreatment of the SR Ca^2+^ channel blockers and the antioxidants. These results demonstrate that doxorubicin could induce ROS generation of cardiomyocytes, thus resulting in an increase in intracellular Ca^2+^ and that the increased intracellular Ca^2+^ further induces ROS production of cardiomyocytes (Kim et al., 2006).

**FIGURE 4:**
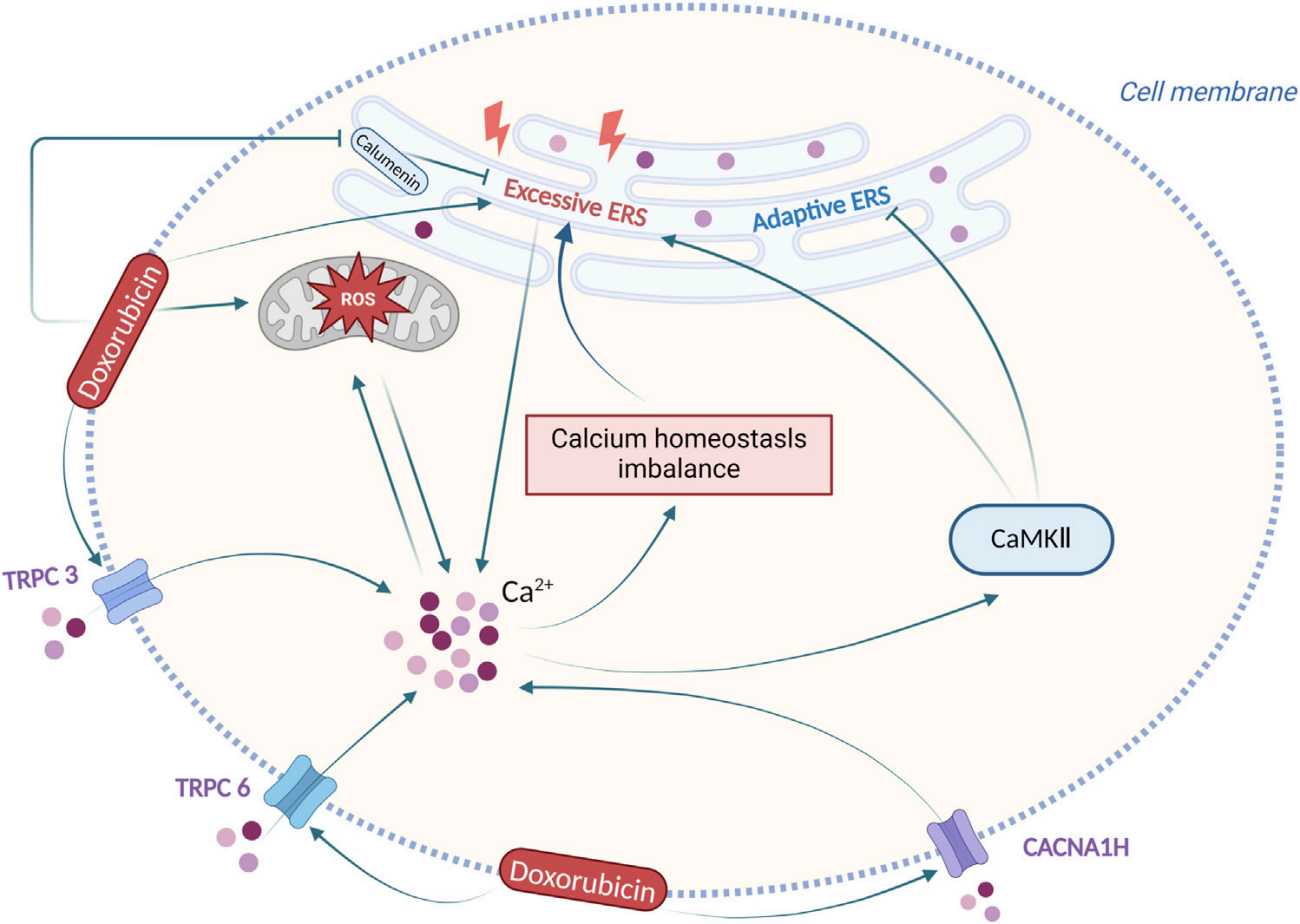
Interaction between ERS and calcium homeostasis imbalance in doxorubicin-induced cardiotoxicity. Produced using BioRender.com (www.biorender.com). Following doxorubicin stimulation, the ERS and the calcium homeostasis imbalance are produced, and altered calcium ion concentration has also a complex effect on ERS.

In conclusion, following doxorubicin-induced ERS, the calcium homeostasis imbalance is produced, and altered calcium ion concentration has also a complex effect on the role of ERS, with the participation of multiple calcium-dependent signalling pathways. Regulation of calcium homeostasis imbalance and ERS could be an effective solution for doxorubicin-induced cardiotoxicity. Figure 4 summarizes the interaction between ERS and calcium homeostasis imbalance in doxorubicin-induced cardiotoxicity.

### 3.3 ERS and autophagy

Autophagy, a process of degradation of damaged cellular components and macromolecules as well as the recycling of catabolic products, is crucial for maintaining intracellular homeostasis (Parzych and Klionsky, 2014). Studies have shown that doxorubicin leads to cell death by affecting the expression of autophagy-related genes to initiate excessive autophagy while restoring normal autophagy protects the heart from doxorubicin-induced cardiotoxicity (Zhu et al., 2009; Bartlett et al., 2016; Li et al., 2016; He et al., 2021). However, the underlying mechanism of autophagy in the development of doxorubicin-induced cardiotoxicity remains unclear. Autophagy is closely related to ERS response. On one hand, autophagy facilitates the restoration of ER homeostasis by eliminating accumulated unfolded and misfolded proteins from ER (Senft and Ronai, 2015). On the other hand, ERS can trigger two types of autophagy: ERS-mediated autophagy and ER-phagy (Song et al., 2018). The UPR signalling pathway is a key promoter in ERS-mediated autophagy. Receptor-mediated selective ER-phagy is initiated by multiple ER surface proteins including FAM134B, RTN3L, TEX264, and ATL3 through the LC3/GABARAP interaction region (LIR/GIM) (Liang et al., 2020). It is worth noting that ERS-associated autophagy is strongly correlated with diseases like non-alcoholic fatty liver disease (NAFLD), vascular calcification, and disc degeneration (Lebeaupin et al., 2018; Huang et al., 2022b; Rao et al., 2022).

In recent years, researchers have also been focusing on the role of ERS-mediated autophagy in doxorubicin-induced cardiotoxicity. One study investigating the impact of interleukin-12p35 on doxorubicin-induced cardiotoxicity reveals that interleukin-12p35 knockdown escalates cardiac injury in mice treated with doxorubicin, along with the occurrence of ERS and autophagy pathway activation. Conversely, mice with doxorubicin-induced cardiotoxicity were treated with interleukin-12 or interleukin-35 experienced diminished cardiac injury along with inhibited autophagy pathways and ERS, suggesting that ERS and autophagy are implicated in the pathogenesis of doxorubicin-induced cardiotoxicity, and interleukin-12p35 might be a contributor to the suppression of ERS and autophagy pathways (Ye et al., 2018). Apart from this, another study found that both FAM134B-mediated ER-phagy and non-FAM134B-mediated autophagy are involved in doxorubicin-induced cardiotoxicity. In an *ex vivo* doxorubicin-induced cardiotoxicity model, Qu et al. found that the overexpression of gasdermin D (GSDMD), a pyroptosis-inducing agent, promoted FAM134B-mediated ER-phagy, leading to the death of cardiomyocytes and worsening of myocardial damage. In contrast, the opposite effect was observed upon inhibition of GSDMD, indicating that FAM134B-mediated ER-phagy is involved in the pathogenesis of doxorubicin-induced cardiotoxicity, and inhibiting GSDMD can reverse the effect (Qu et al., 2022). In addition, scientists found cell-cycle progression gene 1 (CCPG1) -mediated ER-phagy could protect cardiomyocytes from ER stress induced by doxorubicin (Nakagama et al., 2023). Nevertheless, Xu et al. examined the protective effect of Rg1 against doxorubicin-induced cardiac injury in mice, concluding that the protective impact of Rg1 is accomplished by ERS-mediated autophagy (Xu et al., 2018). To summarize, ERS-induced autophagy encourages the pathogenesis of doxorubicin-induced cardiotoxicity, while GSDMD, interleukin-12p35, and Rg1 may affect such a process.

### 3.4 ERS and ubiquitination

Ubiquitin, a small protein molecule comprised of 76 amino acids, undergoes ubiquitination through the action of E1, E2, and E3 ubiquitin ligases, thereby taking part in the regulation of several cellular processes, such as gene transcription, cell cycle progression, and apoptosis (Shaid et al., 2013). Ubiquitination plays a significant role in tumors, neurodegenerative diseases, myasthenia gravis, diabetes, and also doxorubicin-induced cardiotoxicity (Popovic et al., 2014; Wang et al., 2022a). One study indicated that UPR could potentially regulate the ubiquitin-proteasome pathway (Schiattarella et al., 2021). However, other studies detected an elevated level of ERS proteins with impaired ubiquitination (Gerakis et al., 2019; Liu et al., 2020). These findings point to a reciprocal relationship between ubiquitination and ERS. As above, ERS is a significant risk factor in doxorubicin-induced cardiotoxicity (Renu et al., 2018). Ubiquitination may also be involved in doxorubicin-induced cardiotoxicity. In one study, mice suffering doxorubicin-induced cardiotoxicity exhibited elevated levels of E3 ligases, MuRF1 and MAFbx, followed by increased proteasomal degradation markers, and a significant rise in ERS levels, suggesting that doxorubicin could increase ERS by promoting ubiquitination, thereby contributing to cardiotoxicity (Sishi et al., 2013). Another study discovered that inhibiting ubiquitin ligase tripartite motif 25 (TRIM25) in doxorubicin-induced cardiomyocytes led to a decrease of XBP1 nuclear translocation, which increased ERS and exacerbated cardiac injury. Additionally, doxorubicin-treated mice with TRIM25 overexpression developed cardioprotective effects and improved ERS while weakened cardioprotective effects after being injected with the ER stress inducer. It was demonstrated that TRIM25 could inhibit doxorubicin-induced ERS in cardiomyocytes by affecting the activity of the p85α-XBP1 axis through ubiquitination of p85α (Shen et al., 2022), which has proved that ubiquitination has something to do with ERS that contributes to cardiotoxicity caused by doxorubicin.

In summary, doxorubicin-induced cardiotoxicity does have some association with ubiquitination-promoted ERS, and regulating the ubiquitin ligases and their downstream pathways serves as a latent way of intervening doxorubicin-induced cardiotoxicity.

### 3.5 ERS and apoptosis

Apoptosis is a genetically regulated process of programmed cell death crucial in internal environment homeostasis maintaining and proper functioning of organisms. It is categorized into three pathways: the endogenous pathway (mitochondrial pathway), the exogenous pathway (death receptor pathway), and the ERS-induced apoptosis pathway (Hu et al., 2018). Among them, the ERS-induced apoptosis pathway contributes to various diseases, including liver injury (Zhang et al., 2022b), pulmonary fibrosis (Burman et al., 2018), and neurodegenerative disorders (Ghemrawi and Khair, 2020). In doxorubicin-induced cardiotoxicity, ERS also plays a role in cardiac injury by initiating apoptosis (Yarmohammadi et al., 2021).

#### 3.5.1 ERS induces apoptosis through the CHOP pathway

CHOP belongs to the family of CCAAT/enhancer-binding proteins (C/EBPs), regulating cell proliferation, differentiation, and energy metabolism (Ubeda et al., 1996), whereas it can also be regulated by ERS to induce apoptosis through the PERK, ATF6, and IRE1 pathways (Hu et al., 2018). The PERK/eIF2α/ATF4 signalling pathway predominates in the induction of CHOP (Yarmohammadi et al., 2021). CHOP enhances apoptosis mainly through two pathways. One is to upregulate pro-apoptotic proteins such as Bim and downregulate the anti-apoptotic protein Bcl-2. The other is to promote hyperoxidation of the intracellular environment via ERS oxidase 1α(ERO1α) (Tabas and Ron, 2011). During the onset of doxorubicin-induced cardiotoxicity, ERS increases the expression of CHOP through the PERK/eIF2α/ATF4 pathway, thus regulating the levels of apoptotic protein to promote apoptosis.

In one study, researchers found that doxorubicin-induced the expression of CHOP along with an enhancement of apoptosis in isolated cardiomyocytes, indicating ERS-induced apoptosis was involved in doxorubicin-induced cardiotoxicity (Bagchi et al., 2021). Another study found that in cardiomyocytes treated by doxorubicin, overexpression of stress-inducible protein insulin-like growth factor II receptor α (IGF-IIRα) could result in their apoptosis followed by enhanced ERS and induced CHOP. Nevertheless, ERS-mediated apoptosis was attenuated with increasing doses of CHOP-targeted siRNA (Pandey et al., 2022). This suggests that ERS induces apoptosis by increasing CHOP in doxorubicin-induced cardiotoxicity, and IGF-IIRα could initiate this process.

The mechanism of the increase in CHOP expression in doxorubicin-induced cardiomyocytes was further analysed. Zhu et al. (2021b) found that doxorubicin-treated mice developed impaired cardiac function with increased expression of p-PERK, p-eIF2α, ATF4, and CHOP, and the knockdown of stim1 could further aggravate this condition, leading to cardiac cell apoptosis. Kim et al. (2022b) discovered that there was a rise in ATF4 and CHOP expression along with impaired cardiac function in doxorubicin-treated mice, whereas PRMT1 overexpression led to the modification of ATF4, decreased CHOP expression, and a relief in the heart. Another study focused on the role of general control nonderepressible 2 (GCN2), a serine/threonine kinase responsible for the phosphorylation of eIF2α, and found that GCN2 knockdown in doxorubicin-treated mouse cardiomyocytes resulted in an inhibition of ATF4 and CHOP through eIF2α phosphorylation suppression, and restored cardiac function (Wang et al., 2018b).

All of the above has demonstrated that ERS promotes apoptosis by facilitating CHOP expression through the PERK/eIF2α/ATF4 pathway in doxorubicin-induced cardiotoxicity, and modulating PERK/eIF2α/ATF4 pathway may become a new intervention modality for doxorubicin-induced cardiotoxicity.

#### 3.5.2 ERS induces apoptosis through the Caspase-12 pathway

Belonging to an inflammatory caspase, caspase-12 is a protein encoded by the human CASP12 gene that has shown a correlation with ERS-mediated apoptosis (Sahoo et al., 2023). The underlying mechanisms indicated that ERS could activate caspase-12 through an interaction between pro-caspase-12 and IRE1α via the bridging molecule TRAF2. In this way, pro-caspase-12 dissociates from TRAF2 and becomes caspase-12. Another mechanism of pro-caspase-12 activation is through the activation of m-calpain by Ca^2+^ released from ER during ERS. M-calpain is transferred from the cytoplasm into ER to cleave the structural domain of the caspase-recruitment domain (CARD) precursor in caspase-12 and activate it (García de la Cadena and Massieu, 2016). Upon activation, caspase-12 is translocated from ER to the cytoplasm to cleave the caspase-9 precursor, activating the effector caspase-3 and inducing apoptosis (Szegezdi et al., 2003).

In one study, doxorubicin-induced increased expression of ERS markers and chemoattractant receptor-homologous molecule expressed on T helper type 2 cells (CRTH2) in mouse cardiomyocytes, while CRTH2 knockdown could improve the survival rate of doxorubicin treated mice, decrease caspase-12 and caspase-3 levels and reduce apoptosis. In addition, blockage of m-calpain prevented the CRTH2-mediated effect, suggesting that ERS possibly promotes apoptosis through the m-calpain/caspase-12 signalling pathway and CRTH2 could regulate this process (Zuo et al., 2018). In addition, IGF-IIRα might contribute to ERS induction and promote cardiomyocyte apoptosis upon the stimulation of doxorubicin by caspase-12 (Pandey et al., 2022). Other studies found that an increase of caspase-12 as well as some ERS-related proteins in doxorubicin-treated cardiomyocytes, whereas Resolvin D1, interleukin-10 and Shengmai injections ameliorated the doxorubicin-induced apoptosis, along with a significant reduction in both ERS and caspase-12 levels (Chen et al., 2015; Wang et al., 2021; Malik et al., 2022). These suggest ERS induces apoptosis through caspase-12 in doxorubicin-induced cardiotoxicity, and the underlying mechanism of its activation is probably related to Ca^2+^-mediated m-calpain activation. In addition to the above mechanisms, doxorubicin-induced ERS can also be involved in caspase-dependent apoptosis by activating Ca^2+^/calmodulin-dependent protein kinase II (CaMKII) (Tscheschner et al., 2019).

**FIGURE 5:**
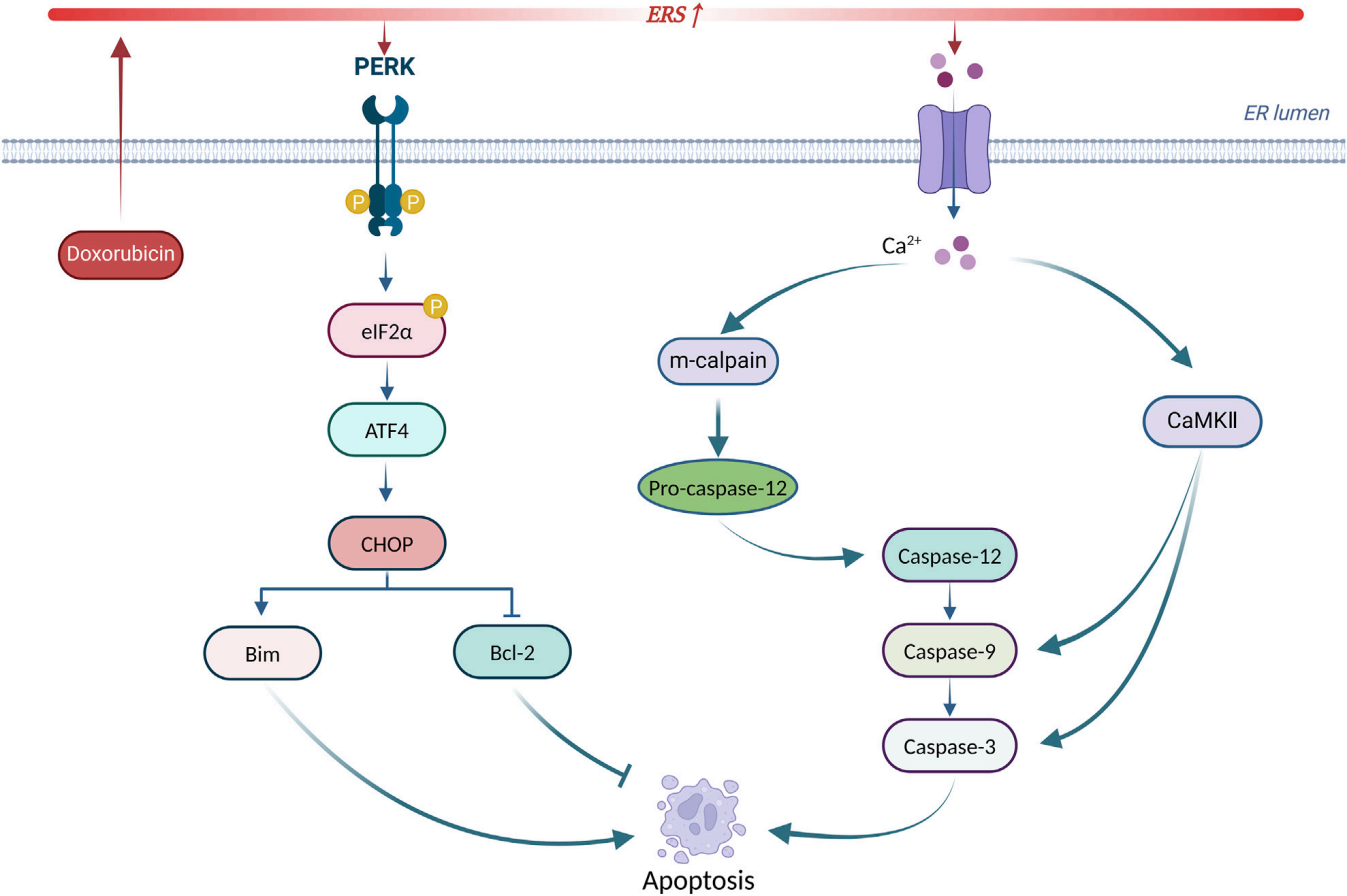
Principal mechanisms of ERS-induced apoptosis in doxorubicin-induced cardiotoxicity. Produced using BioRender.com (www.biorender.com). Doxorubicin activates ERS, leading to UPR receptor activation and calcium efflux. On the one hand, the PERK/eIF2α/ATF4 pathway increases CHOP expression to initiate apoptosis. On the other hand, calcium efflux activates the caspase-dependent apoptotic pathway via m-calpain leading to apoptosis. In addition, calcium efflux activates CaMKII to trigger apoptosis.

In summary, ERS is involved in doxorubicin-induced cardiotoxicity by promoting apoptosis through CHOP, caspase-12, and CaMKII. Targeting ERS-induced apoptosis may serve as a new intervention to treat doxorubicin-induced cardiotoxicity. Figure 5 summarizes the principal mechanisms of ERS-induced apoptosis in doxorubicin-induced cardiotoxicity.

## 4 Targeting ERS for doxorubicin-induced cardiotoxicity

### 4.1 Targeting ERS by clinical drugs

The above has outlined the contribution of ERS to doxorubicin-induced cardiotoxicity, suggesting a putative target of ERS for certain interventions. Researchers have found a variety of clinical drugs that could affect doxorubicin-induced cardiotoxicity by modulating ERS. For example, Sacubitril/valsartan, Folic acid, Doxycycline, Dapagliflozin, Empagliflozin, and Diacerein could reduce ERS and apoptosis, thus inhibiting doxorubicin-induced cardiotoxicity (Lai et al., 2010; Chang et al., 2021; Ye et al., 2021; Kim et al., 2022a; El-Gohary et al., 2024; Malik et al., 2024). Astragalus injection might inhibit doxorubicin-induced excessive ERS by raising calumenin levels and inhibiting phosphorylation of connexin 43 (Cx43) (Ma et al., 2016; He et al., 2018). Moreover, creatine phosphate disodium salt could also attenuate ERS-mediated apoptosis and cardiotoxicity by regulating calumenin (Miao et al., 2016; Wang et al., 2018c). These studies have shed light on novel ways of using those conventional drugs to inhibit doxorubicin-induced cardiotoxicity.

### 4.2 Targeting ERS by chemical compounds

Several newly discovered chemical compounds targeting ERS have been examined the activity against doxorubicin-induced cardiotoxicity recently. For instance, 1910, Resolvin D1 and Sodium hydrosulfide (NaHS) may inhibit doxorubicin-induced cardiotoxicity by modulating CHOP-mediated apoptosis (Lu et al., 2011; Wang et al., 2012b; Wang et al., 2021). 4-phenylbutyrate (4-PBA), Pifithrin-α and Resolvin D1 could also regulate ERS-induced apoptosis through caspase-12, thus attenuating doxorubicin-induced cardiotoxicity (Chua et al., 2006; Fu et al., 2016; Wang et al., 2021).1910, Resolvin D1, and NaHS may also attenuate doxorubicin-induced apoptosis by regulating GRP78 (Lu et al., 2011; Wang et al., 2012b; Wang et al., 2021).

### 4.3 Targeting ERS by phytochemicals

In addition, many phytochemicals have been extracted and utilized to alleviate doxorubicin-induced cardiotoxicity. For example, total flavonoids of Selaginella tamariscina (P. Beauv.) Spring, Ononin, Salvianolic acid B, Resveratrol, Ophiopogonin D and Alginate Oligosaccharide may alleviate doxorubicin-induced cardiotoxicity by modulating CHOP (Meng et al., 2014; Lou et al., 2015; Chen et al., 2016; Guo et al., 2016; Chen et al., 2017; Zhang et al., 2022a; Gao et al., 2022). Similarly, Caffeic acid phenethyl ester may alleviate doxorubicin-induced cardiotoxicity by inhibiting ERS-associated degradation (Zhang et al., 2022c). Apart from the above effects on apoptosis, another compound, Ginsenoside Rg1 may improve doxorubicin-induced cardiac damage by inhibiting ERS and autophagy (Xu et al., 2018). Additionally, Bergamot Polyphenolic Fraction, Olea europea L. Extract, and Cynara cardunculus may reduce doxorubicin-induced cardiotoxicity by modulating ATF6 (Maiuolo et al., 2021). Biotin-conjugated ADTM analog (BAA) may attenuate doxorubicin-induced apoptosis by regulating GRP78 (Zhang et al., 2019).

### 4.4 Targeting ERS by microRNAs

Apart from the above-mentioned, non-coding RNAs especially microRNAs also show a potential to manage ERS and reduce doxorubicin-induced cardiotoxicity. miR-378 plays a crucial role in various cancers (Zeng et al., 2017), and it also plays a key role in doxorubicin-induced cardiotoxicity by regulating ERS-induced apoptosis. The underlying mechanisms indicate that miR-378 regulates cyclophilin A (PPIA), a regulator of protein folding (Nigro et al., 2013), to restrain the PERK-CHOP pathway and inhibit doxorubicin-induced cardiotoxicity (Wang et al., 2018d). Another study demonstrated that miR-378 could increase the level of calumenin to mitigate ERS induced by calcium homeostasis imbalance, thus alleviating doxorubicin-induced cardiotoxicity. Moreover, Huang et al. (2022a) discovered that miR-181a-5p, another microRNA whose content could be improved by intravenous injection of mesenchymal stromal cells, reduced ERS-induced apoptosis in doxorubicin treated cardiomyocytes via inhibiting GRP78. In addition, scientists found inhibition of miR-194-5p could alleviate doxorubicin-induced cardiotoxicity via P21-activated kinase 2 (PAK2) and XBP1s, and miR-194-5p/PAK2/XBP1s axis might be the potential targets for doxorubicin-induced cardiotoxicity (Fa et al., 2022).

**FIGURE 6:**
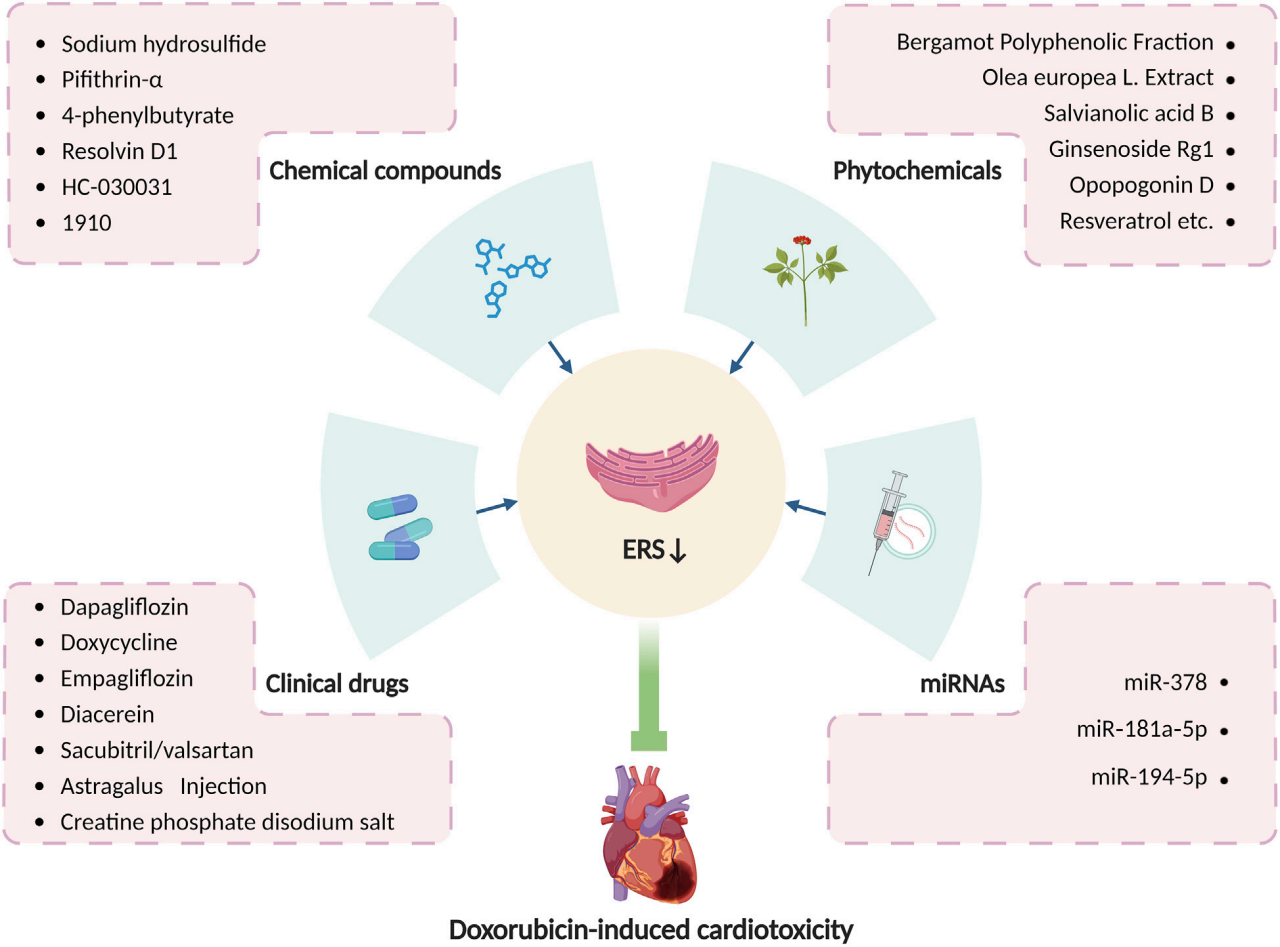
Interventions targeting ERS in doxorubicin-induced cardiotoxicity. Produced using BioRender.com (www.biorender.com). Many clinical drugs, chemical compounds, phytochemicals, and some microRNAs can ameliorate doxorubicin-induced cardiotoxicity by targeting ERS.

In summary, various medications have been proven accessible to relieve doxorubicin-induced cardiotoxicity by altering ERS, and modulation of ERS may serve as a new target for the treatment of doxorubicin-induced cardiotoxicity. With consistently advanced and updated science and technology, more pieces of the puzzle of ERS in doxorubicin-induced cardiotoxicity will be found, which will provide a brighter future in the treatment of doxorubicin-induced cardiotoxicity by targeting ERS. Table 1 summarizes principal compounds targeting ERS to improve doxorubicin-induced cardiotoxicity, and Figure 6 summarizes the interventions targeting ERS in doxorubicin-induced cardiotoxicity.

**TABLE 1:** Principal compounds targeting ERS to improve doxorubicin-induced cardiotoxicity.

Category	Drug name	Model (Animals/Cells)	Doxorubicin dose/route of administration	Drug dose/route of administration	Change of ERS markers	Main effect	Reference
Chemical compounds	Pifithrin-α	H9c2	5 μmol/L for 8 h or 16 h	20 μmol/L for 8 h or 16 h	Caspase-12↓	Apoptosis↓	Chua et al. (2006)
	1910, Rogersitib or Estybon	Female Nude mice	2.75 mg/kg/q2d or 1.75 mg/kg/q2d; i.p	200 mg/kg/q2d; i.p	GRP78↓ CHOP↓	Body weight↑ Cardiac weight↑ Cardiac tissue damage↓	Lu et al. (2011)
	Sodium hydrosulfide	H9c2	5 μmol/L for 24 h	400 μmol/L for 0.5 h	GRP78↓ CHOP↓	Oxidative stress↓ Cytotoxicity↓	Wang et al. (2012b)
	4-phenylbutyrate	Male ICR mice NRCM	15 mg/kg; i.p 1 μmol/L for 24 h	100 mg/kg/d for 7 days; i.p 0.5 mmol/L	Caspase-12 cleavage↓	Cardiac dysfunction↓ Apoptosis↓	Fu et al. (2016)
	HC-030031	Male C57BL/6J mice	20 mg/kg; i.p	10 mg/kg/d for 10 days; i.g	GRP78↓ CHOP↓ ATF6↓ p-eIF2↓ XBP-1↓ Caspase-12↓	Oxidative stress↓ Inflammation↓ Apoptosis↓	Wang et al. (2018e)
	Resolvin D1	Male C57BL/6 mice	20 mg/kg; i.p	2.5 μg/kg/d; i.p	p-PERK↓ GRP78↓ p-eIF2α↓ CHOP↓ ATF6↓ Caspase-12↓	Apoptosis↓ Oxidative stress↓ Cardiac dysfunction↓ Inflammation↓	Wang et al. (2021)
Phytochemicals	Ophiopogonin D	Male C57BL/6 mice H9c2	2 mg/kg/q2d; i.p 1 μmol/L for 12 h	10 mg/kg/q2d; i.g 1 μmol/L for 12 h	ATF6↓ GRP78↓ CHOP↓	Cardiac ultrastructural abnormalities↓ Cell viability↑ Oxidative stress↓	Meng et al. (2014)
	Resveratrol	H9c2	5 μmol/L for 24 h	25 μmol/L for 24 h	CHOP↓ GRP78↓	Apoptosis↓ Cell viability↑	Lou et al. (2015)
	Salvianolic acid B	Male BALB/c mice	20 mg/kg; i.p	2 mg/kg/d for 7 days; i.v	GRP78↓ CHOP↓ p-IRE-1↓ ATF6↓ p-PERK↓	Body weight↓ Cardiac dysfunction↓ Cardiac damage↓ Apoptosis↓	Chen et al. (2016)
	Alginate Oligosaccharide	Male C57BL/6 mice	20 mg/kg; i.p	200 mg/kg/d for 7 days; i.g	CHOP↓ Caspase-12↓	Cardiac dysfunction↓ Mortality↓ Heart-to-body weight ratio↑ Cardiac injury↓ Apoptosis↓ Oxidative stress↓	Guo et al. (2016)
	Salvianolic acid B	Male SD rats ARVM	3 mg/kg/q2d for 3 times; i.p 1 μmol/L for 4 h	0.25, 0.5, or 1 mg/kg/d for 7 days; i.v 20 μg/mL for 6 h	GRP78↓ CHOP↓	Cardiotoxicity↓ Apoptosis↓	Chen et al. (2017)
	Ginsenoside Rg1	Male C57BL/6 mice	6 mg/kg/q3d for 4 times; i.p	50 mg/kg/d	ATF6↓ IRE1↓ XBP1u↓ GRP78↑	Cardiac function↑ Cardiac fibrosis↓ Cardiac autophagy↓	Xu et al. (2018)
	Biotin-conjugated ADTM analog	H9c2	1 μmol/L for 24 h	10 μmol/L, 30 μmol/L, or 100 μmol/L for 24 h	GRP78↓ p-eIF2α↓	Cytotoxicity↓	Zhang et al. (2019)
	Bergamot Polyphenolic Fraction Oleuropein Cynara cardunculus	H9c2	1 μmol/L for 6 h	5 μg/mL for 24 h 1 μmol/L for 24 h 1 μmol/L for 24 h	ATF6↓	Apoptosis↓ Cell viability↑ Oxidative damage↓	Maiuolo et al. (2021)
	Total flavonoids of Selaginella tamariscina (P.Beauv.) Spring	Male C57BL/6 mice H9c2	5 mg/kg/d for 4 days; i.p 1 μmol/L for 24 h	200 μL/10 g; i.g 10 μg/mL for 24 h	PERK↓ ATF4↓ CHOP↓	Myocardial malfunction↓ Cardiac injury↓ Oxidative stress↓ Mitochondrial dysfunction↓ Apoptosis↓	Gao et al. (2022)
	Ononin	Male Wistar rats	2.5 mg/kg/q7d for 6 times; i.v	30 or 60 mg/kg/d for 8 weeks; i.g	CHOP↓ GPR78↓	Cardiac tissue damage↓ Cell viability↑ Apoptosis↓	Zhang et al. (2022a)
	Caffeic acid phenethyl ester	H9c2	1 μmol/L for 24 h	10 μmol/L for 24 h	p-elF2α↓ ERp29↓ Bap31↓ ERp57↓ Sec61γ↓	Cytotoxicity↓	Zhang et al. (2022c)

*eIF2α*, eukaryotic initiation factor-2α, *GRP78*, Glucose-regulated protein 78, *CHOP*, C/EBP homologous protein; *PERK*, Protein kinase R (PKR)-like ER, kinase; *ATF6*, Activating transcription factor 6, *H9c2* Rat embryonic cardiomyoblasts, *ERS*, endoplasmic reticulum stress; *SD*, Sprague-Dawley; *IRE-1*, Inositol-requiring enzyme 1, *ATF4*, Activating transcription factor 4; *i. p*., Intraperitoneal injection; *i. v*., Intravenous injection; *i. g*., Intragastric administration; *XBP1u*, X-box binding protein 1u; *ERp29*, Endoplasmic reticulum protein 29; *ERp57*, Endoplasmic reticulum protein 57; *Bap31*, B-cell receptor-associated protein 31; *IRE-1α*, Inositol-requiring enzyme 1α; *NRCM*, neonatal rat cardiomyocytes; *ARVM*, adult rat ventricular myocytes; *ICR*, institute of cancer research.

## 5 Future and prospect

Current studies suggest that ERS is a crucial factor in the development of doxorubicin-induced cardiotoxicity. Various studies have shown that doxorubicin causes cardiac injury through numerous mechanisms related to ERS, including autophagy, ubiquitination, oxidative stress, and apoptosis, demonstrating a possibility that ERS inhibition may come to an effect in the intervention of doxorubicin-induced cardiotoxicity. As evidence, newly discovered drugs and methods targeting ERS have been proven to reduce doxorubicin-induced cardiotoxicity. However, the ERS-UPR system, which serves as a mechanism for the cell to restore homeostasis, is ubiquitous in cells. Therefore, excessive inhibition of ERS may exacerbate the disease. So, it is of great value to investigate how to regulate it appropriately. In addition, it remains unclear whether ERS inhibitors adversely affect other parts of the human body, which may bring multiple side effects. Therefore, it is important to evaluate the side effects of ERS inhibitors. The present studies mainly focus on the role of ERS in doxorubicin-treated animal cardiac tissues or cardiomyocytes, not the accurate role of ERS in cardiac tissues of patients treated by doxorubicin, resulting in an urgent need for further clinical trials to confirm whether the ERS inhibitor can be used for treatment effectively. Moreover, experimental evidence indicates that the UPR pathway suppresses doxorubicin-induced cardiotoxicity in the early stages, but a conversion from beneficial UPR to the excessive ERS response will lead to cardiac impairment, with a still perplexing mechanism, and how the three UPR pathways activate and interact with each other, need further investigation.

This review has presented an overview of the interaction of ERS with various mechanisms, including autophagy, ubiquitination, oxidative stress, calcium homeostasis imbalance, and apoptosis in the development of doxorubicin-induced cardiotoxicity. Furthermore, different types of potential interventions targeting ERS for doxorubicin-induced cardiotoxicity, including clinical drugs, chemical compounds, phytochemicals as well as miRNA, are also embraced. We are confident that as soon as all speculations turn into certainties, modulation of ERS will show its capabilities in the treatment of doxorubicin-induced cardiotoxicity.
